# Trends and Determinants of Preterm Births

**DOI:** 10.7759/cureus.104467

**Published:** 2026-03-01

**Authors:** Faris Kazic, Enid Nakicevic, Asmira Ibrasevic, Hakija Sejdic

**Affiliations:** 1 Obstetrics and Gynecology, Cantonal Hospital Zenica, Zenica, BIH

**Keywords:** hypertensive disorders, maternal comorbidities, neonatal outcomes, preterm birth, prom, risk factors

## Abstract

Objective

Preterm birth remains a leading cause of perinatal morbidity and mortality. This study aimed to retrospectively analyze the trends in preterm birth rates and investigate key maternal and fetal determinants at the Cantonal Hospital Zenica, a secondary referral center, over a five-year period (2020-2024).

Methods

A retrospective, descriptive-analytical study was conducted that included all preterm deliveries (<37 gestational weeks) at the Department of Gynecology and Obstetrics. Data were collected from official delivery registries. Statistical analysis included linear regression for trends, chi-square tests for categorical variables, and Spearman’s correlation to assess associations between maternal parity, comorbidities (hypertension, obesity, thyroid disorders), mode of delivery, premature rupture of membranes (PROM), and neonatal outcomes (Apgar scores, hospitalization length, and neonatal intensive care unit (NICU) transfer).

Results

Out of 11,117 total deliveries, 423 (3.80%) were preterm. The incidence remained stable across the study period. Multiparous women accounted for 53.19% of cases. The most prevalent comorbidities were obesity (27.4%) and hypertensive disorders (16.1%). A significant association was found between hypertensive disorders and delivery by cesarean section (73.53%; p<0.005), whereas these mothers had a significantly lower incidence of PROM. Conversely, primiparity was significantly associated with a higher rate of PROM (52.75%; p = 0.0115). Vaginal delivery was more frequent in cases with PROM (64.68%). Neonatal hospitalization showed a decreasing trend (R² = 0.7156). Transfer to a tertiary NICU was significantly correlated with lower gestational age and lower Apgar scores.

Conclusion

The incidence of preterm birth in the observed region is stable and relatively low. The study highlights two distinct patterns of preterm delivery: spontaneous preterm labor associated with PROM and vaginal delivery in primiparous women, versus medically indicated preterm delivery driven by maternal comorbidities like hypertension and obesity, resulting in higher cesarean section rates. Effective antenatal management of these comorbidities is crucial for improving perinatal outcomes.

## Introduction

Preterm birth, defined as delivery before 37 completed weeks of gestation, remains a significant global public health challenge. Despite advances in perinatal care, approximately 15 million infants are born preterm annually, accounting for more than one in 10 newborns [1]. The clinical significance of preterm birth lies not only in its high prevalence but also in the substantial risk of perinatal mortality and long-term morbidity, including respiratory complications and neurodevelopmental impairments. Furthermore, the economic burden on healthcare systems is considerable due to the intensive neonatal care required. The etiology involves a complex interplay of maternal demographic factors, obstetric history, lifestyle choices, and underlying pathologies, necessitating a nuanced understanding to develop effective preventive strategies [2,3].

A critical aspect of preterm birth etiology involves the distinction between spontaneous and medically indicated deliveries. Specific maternal conditions such as hypertensive disorders of pregnancy [4,5], thyroid dysfunction [6], and obesity [7,8] play pivotal roles, frequently necessitating iatrogenic preterm delivery to safeguard maternal and fetal health. Conversely, premature rupture of membranes (PROM) represents a primary pathway for spontaneous preterm birth, often driven by infection or inflammation [9,10]. Understanding the distinct contributions of these factors, whether leading to spontaneous labor or mandated intervention, is essential for comprehensive management.

The present study, therefore, aimed to retrospectively analyze the trends in preterm birth rates and investigate key maternal and fetal determinants at the Cantonal Hospital Zenica, a secondary referral center, over a five-year period (2020-2024). Specifically, this research sought to identify factors most strongly associated with preterm birth risk and assess whether the profile of high-risk pregnant women has changed during the observed period.

## Materials and methods

This study was designed as a retrospective, descriptive, analytical review of delivery records and medical documentation from the Department of Gynecology and Obstetrics at the Cantonal Hospital Zenica. The study encompassed a five-year period, from January 1, 2020, to December 31, 2024. As a secondary referral center providing comprehensive obstetric care for the Zenica-Doboj Canton, it is important to note that the hospital does not operate its own neonatal intensive care unit (NICU). Consequently, preterm neonates requiring intensive care are routinely transferred to the tertiary referral center's NICU at the University Clinical Center in Sarajevo for specialized treatment.

Data were meticulously collected from official delivery registries and individual patient medical records routinely maintained at the department. The study population comprised all women who delivered neonates diagnosed as preterm (<37 completed weeks of gestation) at the Cantonal Hospital Zenica during the specified period. For analytical purposes, maternal characteristics were recorded once per delivery event, while neonatal outcomes were extracted for each infant. Deliveries with incomplete or illegible medical records, cases with unreliably determined gestational age, induced abortions, therapeutic terminations of pregnancy, and stillbirths were excluded. Systematically extracted variables pertinent to preterm birth determinants and outcomes included maternal parity (primiparous or multiparous), maternal age, gestational age at delivery, fetal sex, neonatal birth weight, Apgar scores at one minute and five minutes, presence of maternal chronic conditions, PROM, duration of neonatal hospital stay postpartum, and eventual transfer of the neonate to a NICU. Gestational age at delivery was further categorized into clinically relevant groups: 35-36+6 weeks, 33-34+6 weeks, and <33 weeks. These categories represent operational definitions used within our study, reflecting the local clinical practice and data stratification relevant for management and referral decisions at our secondary referral hospital.

Statistical analysis was performed using descriptive and inferential methods. Descriptive statistics included means, standard deviations (SD), minimum and maximum values for continuous variables, and absolute/relative frequencies for categorical variables. Annual and overall preterm delivery incidences were expressed as percentages of total deliveries. Temporal trends in preterm birth rates and neonatal hospitalization duration were examined using simple linear regression, yielding regression coefficients and coefficients of determination (R²). Differences in categorical variable distributions were evaluated using chi-square (χ²) tests. Mean continuous variables were compared across study years using one-way analysis of variance (ANOVA) and between the two groups using independent-samples t-tests. Associations between variables were assessed with Spearman's rank correlation coefficient (r). All statistical tests were two-tailed, with p < 0.05 considered statistically significant.

## Results

During the observed period from 2020 to 2024, there were a total of 11,117 deliveries. The average incidence of preterm births was 423 out of 11,117 (3.80%). The percentage of preterm births was consistent across years. These data are presented in Table 1.

**Table 1:** Incidence of preterm births relative to total deliveries in the period 2020-2024 Preterm births: all preterm births for the displayed year and the percentage of preterm births in a given year relative to the total number of deliveries in that year; ∑: sum of data from the specified rows

Year	Preterm births		Total number of deliveries
2020	100	4.04%	2478
2021	73	3.21%	2277
2022	93	4.30%	2161
2023	66	3.17%	2083
2024	91	4.30%	2118
∑	423	3.80%	11117

The average proportion of primiparous women among preterm births was 198 out of 423 (46.81%). The most significant difference was observed in 2021 at 57.53%, while the smallest was in 2022 at 39.78%. The observed differences in the percentages of primiparous versus multiparous women varied from year to year but were neither substantial nor statistically significant (χ² = 6.9019; p < 0.1411; df = 4; χ²sig = 9.4877). These data are presented in Table 2.

**Table 2:** Maternal parity distribution by year Primiparous women: number of primiparous women with preterm birth in a given year; multiparous women: number of multiparous women with preterm birth in a given year; ∑: sum of data from the specified rows Data are presented as numbers (percentages). Statistical test used: chi-square test

Year	Primiparous women		Multiparous women		N	Test statistic	p-value
2020	51	51.00%	49	49.00%	100	6.9019	0.1411
2021	42	57.53%	31	42.47%	73		
2022	37	39.78%	56	60.22%	93		
2023	30	45.45%	36	54.55%	66		
2024	38	41.76%	53	58.24%	91		
∑	198	46.81%	225	53.19%	423		

**Figure 1:**
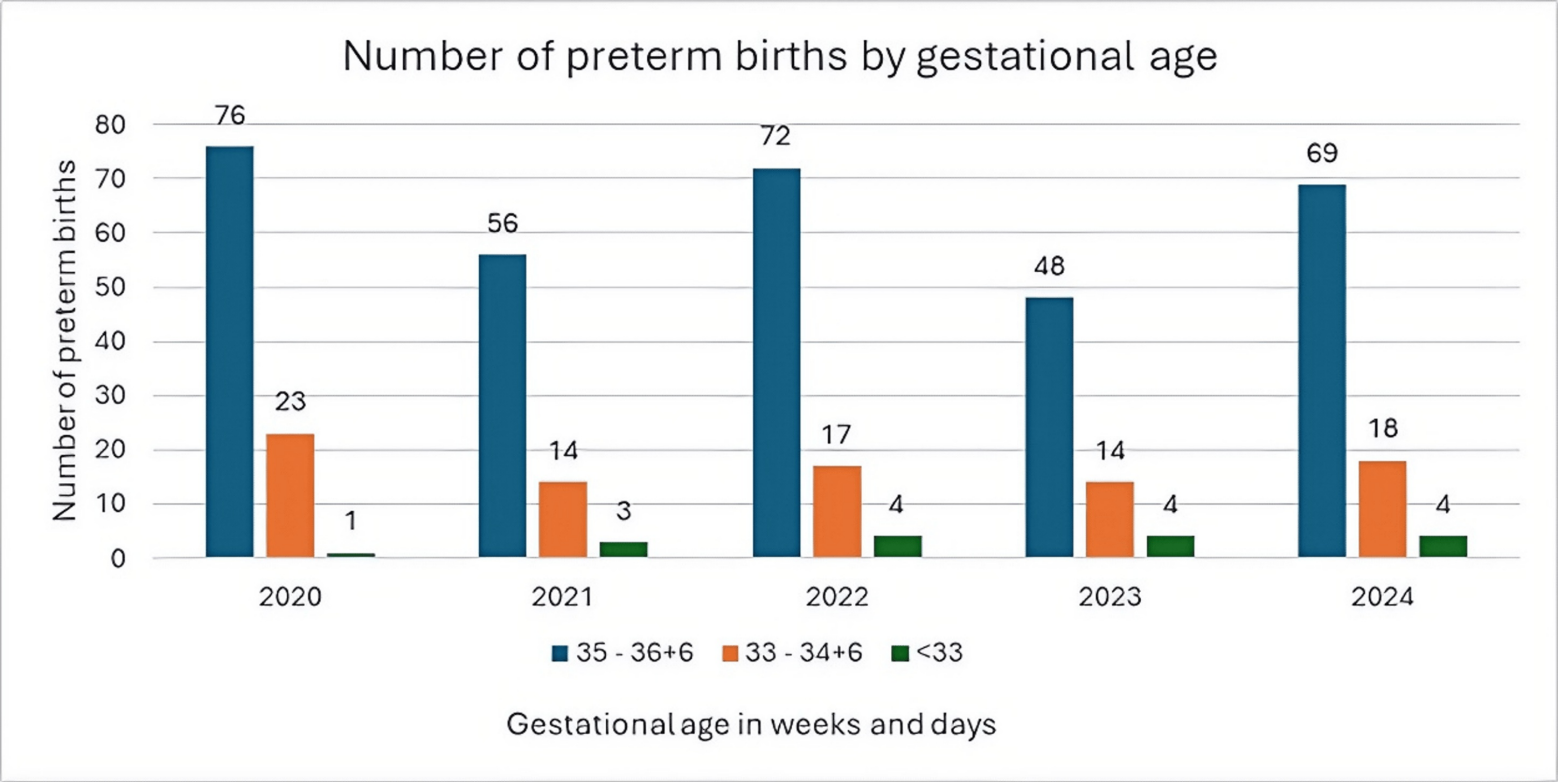
Gestational periods of preterm births

When observing the distribution of maternal parity according to gestational age, a higher percentage of births before 33 weeks of gestation is noted among multiparous women, 12 out of 16 (75.0%), compared to primiparous women, four out of 16 (25.0%); however, this was not statistically significant (χ² = 3.1765; p < 0.0747; df = 1; χ²sig = 3.841). These data are presented in Table 3 and Figure 1.

**Table 3:** Maternal parity distribution by gestational age Primiparous women: total number of primiparous women for a specific gestational group observed across all years included in the study; multiparous women: total number of multiparous women for a specific gestational group observed across all years included in the study; ∑: sum of data from the specified rows Data are presented as numbers (percentages). Statistical test used: chi-square test

Gestational age	Primiparous women		Multiparous women		N	Test statistic	p-value
35-36+6	152	47.4%	169	52.6%	321	3.1765	0.0747
33-34+6	42	48.8%	44	51.2%	86		
<33	4	25.0%	12	75.0%	16		
∑	198	46.8%	225	53.2%	423		

When observing the distribution of infant sex according to gestational age, a higher percentage of male infants, 10 out of 16(62.5%), was noted for births below 33 weeks of gestation, compared to female infants, six out of 16 (37.5%); however, this was not statistically significant (χ² = 0.39333; p = 0.5305; df = 1; χ²sig = 3.841). These data are presented in Table 4.

**Table 4:** Distribution of infant sex by gestational age Male: total number of live-born male infants for a specific gestational group observed across all years included in the study; female: total number of live-born female infants for a specific gestational group observed across all years included in the study; ∑: sum of data from the specified rows Data are presented as numbers (percentages). Statistical test used: chi-square test

Gestational age	Male		Female		N	Test statistic	p-value
35-36+6	173	53.9%	148	46.1%	321	0.39333	0.5305
33-34+6	49	57.0%	37	43.0%	86		
<33	10	62.5%	6	37.5%	16		
∑	232	54.8%	191	45.2%	423		

The lowest average one-minute Apgar score was recorded in 2021 at 7.16 ± 1.4, while the highest average was in 2020 at 7.50 ± 1.08. In 2021 and 2022, the minimum one-minute Apgar score was 1, and the maximum was 9 in both years. A one-minute Apgar score below 8 was most prevalent in 2021 at 37 out of 73 cases (50.68%), and least prevalent in 2020 at 33 out of 100 cases (33.0%), but this difference was not statistically significant (χ² = 7.243; p = 0.12358; df = 4; χ²sig = 9.4877). These data are presented in Table 5.

**Table 5:** Average one-minute Apgar score by year Average: average one-minute Apgar score by year; minimum: minimum one-minute Apgar score by year; maximum: maximum one-minute Apgar score by year; Apgar score <8: total number of Apgar scores less than 8 recorded for a specific year Data are presented as numbers (percentages). Statistical test used: chi-square test comparing the frequency of scores <8 versus ≥8 across years

Year	Average	Standard deviation	Minimum	Maximum	Apgar score < 8		N	Test statistic	p-value
2020	7.50	1.08	3	9	33	33.00%	100	7.243	0.12358
2021	7.16	1.40	1	9	37	50.68%	73		
2022	7.26	1.38	1	9	45	48.39%	93		
2023	7.27	1.22	2	9	31	46.97%	66		
2024	7.30	1.28	2	9	39	42.86%	91		

The lowest average five-minute Apgar score was in 2021 at 8.05 ± 1.17, while the highest average was in 2020 at 8.45 ± 0.82. In 2022 and 2024, the minimum five-minute Apgar score was 2, and the maximum was 10 in all years, except 2021, when it was 9. A five-minute Apgar score below 8 was most prevalent in 2023 at 14 out of 66 cases (21.21%), and least prevalent in 2020 at 10 out of 100 cases (10.0%), but this was not statistically significant (χ² = 5.8847; 0.2079; df = 4; χ²sig = 9.4877). These data are presented in Table 6.

**Table 6:** Average five-minute Apgar score by year Average: average five-minute Apgar score by year; minimum: minimum five-minute Apgar score by year; maximum: maximum five-minute Apgar score by year; Apgar score <8: total number of Apgar scores less than 8 recorded for a specific year Data are presented as numbers (percentages). Statistical test used: chi-square test comparing the frequency of scores <8 versus ≥8 across years

Year	Average	Standard deviation	Minimum	Maximum	Apgar score<8		N	Test statistic	p-value
2020	8.45	0.82	5	10	10	10.00%	100	5.8847	0.2079
2021	8.05	1.17	3	9	15	20.55%	73		
2022	8.22	1.22	2	10	12	12.90%	93		
2023	8.18	1.19	4	10	14	21.21%	66		
2024	8.20	1.16	2	10	15	16.48%	91		

Natural (vaginal) deliveries, accounting for 252 out of 423 (59.6%), are more prevalent than cesarean sections, which account for 171 out of 423 (40.4%) of preterm births. The observed differences between the examined gestational age groups were not statistically significant (χ² = 0.5615; p = 0.7552; df = 2; χ²sig = 5.9914). These data are presented in Table 7.

**Table 7:** Distribution of natural deliveries and cesarean sections by gestational age Natural birth: all preterm natural deliveries for a specific gestational group observed across all years included in the study; cesarean section: all preterm deliveries by cesarean section for a specific gestational group observed across all years included in the study; ∑: sum of data from the specified rows Data are presented as numbers (percentages). Statistical test used: chi-square test

Gestational age	Natural birth		Cesarean section		N	Test statistic	p-value
35-36+6	188	58.6%	133	41.4%	321	0.5615	0.7552
33-34+6	54	62.8%	32	37.2%	86		
<33	10	62.5%	6	37.5%	16		
∑	252	59.6%	171	40.4%	423		

The relationships between maternal parity (primiparous or multiparous) and other statistical groups were investigated using Spearman's correlation. Statistically significant associations were found: a weak correlation was observed between maternal parity and the five-minute Apgar score (r = 0.150; n = 423; p = 0.002), with multiparous women having higher five-minute Apgar scores. A weak negative correlation was observed between maternal parity and the number of hospital days (r = -0.184; n = 423; p = 0.00014), indicating that multiparous women have shorter hospital stays. A weak negative correlation was observed between maternal parity and PROM (r = -0.123; n = 423; p = 0.011), with primiparous women experiencing a higher rate of PROM. These data are presented in Table 8.

**Table 8:** Spearman’s correlation of maternal parity in relation to other statistical groups Statistical test used: Spearman's rank correlation. *Significant at p < 0.05. **Significant at p < 0.01

	Mode	Gestational age	Apgar one minute	Apgar five minutes	Duration of neonatal hospitalization	PROM	NICU
Test statistic (correlation coefficient r)	-0.027	0.036	-0.014	-0.150^**^	-0.184^**^	-0.123*	0.039
p-value	0.580	0.463	0.771	0.002	0.00014	0.011	0.422

Primiparous mothers showed a higher incidence of PROM, occurring in 115 out of 215 (52.75%) of cases, compared to multiparous mothers at 103 out of 218 (47.25%) of cases, which was statistically significant (χ² = 6.382; p = 0.0115; df = 1; χ²sig = 3.8414). These data are presented in Table 9.

**Table 9:** Incidence of premature rupture of membranes according to maternal parity PROM: premature rupture of membranes; ∑: sum of data from the specified rows Data are presented as numbers (percentages). Statistical test used: chi-square test. Significant at p < 0.05

PROM	Primiparous women		Multiparous women		N	Test statistic	p-value
yes	115	52.75%	103	47.25%	218	6.382	0.0115*
No	83	40.49%	122	59.51%	205		
∑	198	46.81%	225	53.19%	423		

**Figure 2:**
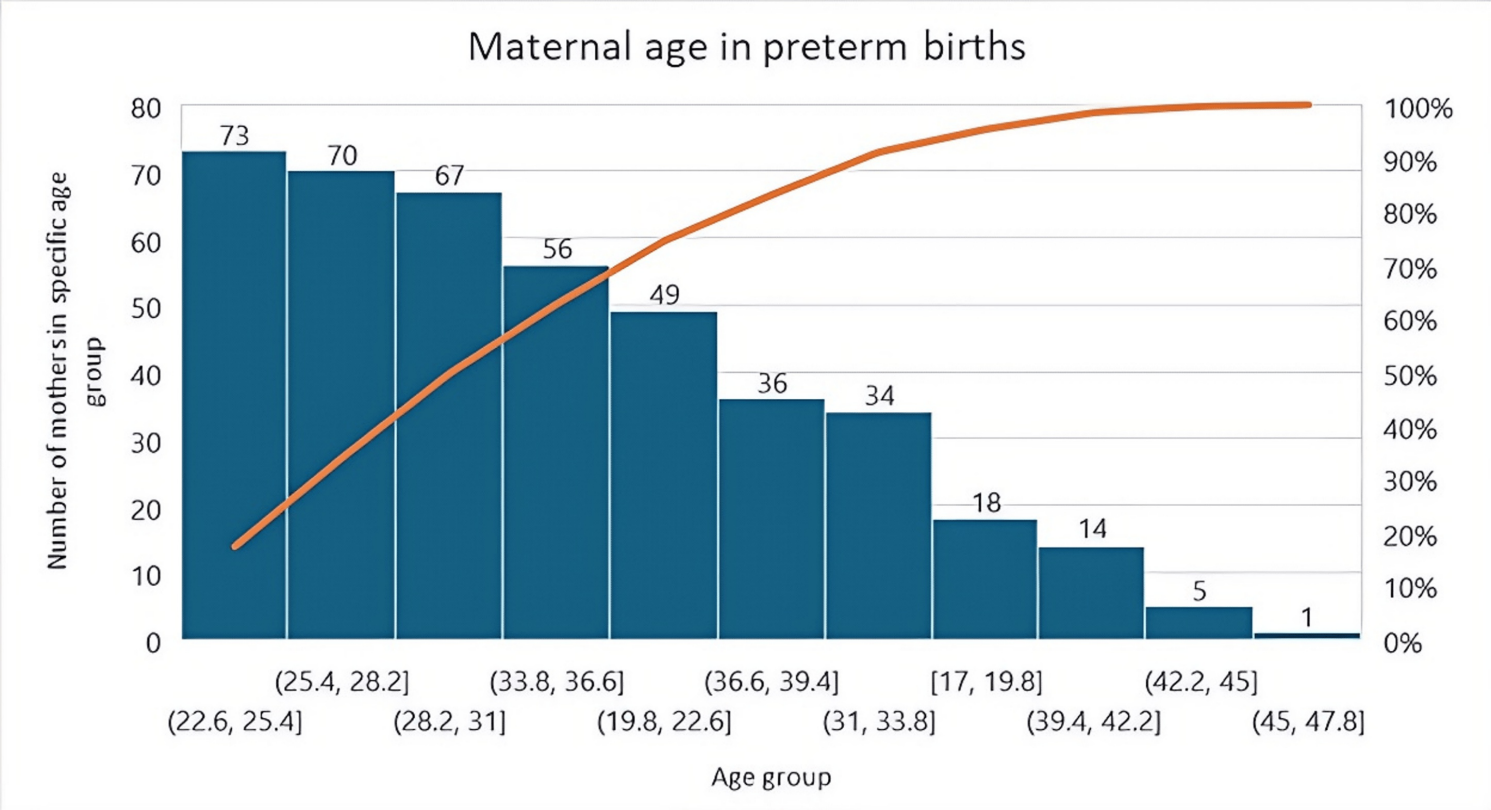
Maternal age in preterm births

The grouped maternal age ranges for women who experienced preterm birth are presented in the graph. The most represented age group, which most frequently experienced preterm birth, was between 22.6 and 25.4 years of age. These data are presented in Figure 2.

Natural (vaginal) deliveries were more prevalent among multiparous women at 138 out of 225 (61.33%), compared to cesarean sections among multiparous women at 87 out of 225 (38.67%). Natural deliveries were also more prevalent among primiparous women at 57.58% compared to cesarean sections among primiparous women, but these differences were not statistically significant (χ² = 0.6174; p = 0.4319; df = 1; χ²sig = 3.8414). These data are presented in Table 10.

**Table 10:** Distribution of mode of delivery in relation to parity ∑: sum of data from the specified rows Data are presented as numbers (percentages). Statistical test used: chi-square test

Parity	Vaginal birth		Cesarean section		N		Test statistic	p-value
Primiparous women	114	57.58%	84	42.42%	198	100.00%	0.6174	0.4319
Multiparous women	138	61.33%	87	38.67%	225	100.00%		
∑	252	59.57%	171	40.43%	423	100.00%		

Natural (vaginal) deliveries were more prevalent in cases with PROM, accounting for 141 out of 218 (64.68%), compared to cesarean sections in cases with PROM at 77 out of 218 (35.32%), which was statistically significant (χ² = 4.8665; p = 0.02738; df = 1; χ²sig = 3.8414). These data are presented in Table 11.

**Table 11:** Distribution of mode of delivery in relation to premature rupture of membranes PROM: premature rupture of membranes; ∑: sum of data from the specified rows Data are presented as numbers (percentages). Statistical test used: chi-square test. Significant at p < 0.05

RVP	Vaginal birth		Cesarean section		N		Test statistic	p-value
Yes	141	64.68%	77	35.32%	218	100.00%	4.8665	0.02738*
No	111	54.15%	94	45.85%	205	100.00%		
∑	252	59.57%	171	40.43%	423	100.00%		

Natural (vaginal) deliveries were more prevalent in cases requiring NICU admission, accounting for 37 out of 64 (57.81%), compared to cesarean sections in cases requiring NICU at 27 out of 64 (42.19%), but this was not statistically significant (χ² = 0.0972; p = 0.7552; df = 1; χ²sig = 3.8414). These data are presented in Table 12.

**Table 12:** Distribution of mode of delivery by NICU transfer NICU: neonatal intensive care unit; ∑: sum of data from the specified rows Data are presented as numbers (percentages). Statistical test used: chi-square test

NICU	Vaginal birth		Cesarean section		N		Test statistic	p-value
Yes	37	57.81%	27	42.19%	64	100.00%	0.0972	0.7552
No	215	59.89%	144	40.11%	359	100.00%		
∑	252	59.57%	171	40.43%	423	100.00%		

The most prevalent comorbidity was obesity, affecting 116 out of 423 (27.4%) of mothers, followed by hypertensive pregnancy disease at 68 out of 423 (16.1%), and thyroid diseases at 62 out of 423 (14.7%). Diabetes was the least prevalent, occurring in 40 out of 423 (9.5%) of cases. These data are presented in Table 13.

**Table 13:** Comorbidities in mothers with preterm births

Comorbidities	Yes		No		N
Hypertensive disorders in pregnancy	68	16.1%	355	83.9%	423
Diabetes mellitus	40	9.5%	383	90.5%	423
Thyroid disorders in pregnancy	62	14.7%	361	85.3%	423
Obesity	116	27.4%	307	72.6%	423

The majority of mothers with preterm births had no comorbidities, accounting for 229 out of 423 (54.1%) of cases. One comorbidity was present in 117 out of 423 (27.7%) of mothers, two comorbidities in 63 out of 423 (14.9%), three comorbidities in 13 out of 423 (3.1%), and all four comorbidities in one mother. These data are presented in Table 14.

**Table 14:** Multiple comorbidities in mothers with preterm births

Number of comorbidities	N	
0	229	54.1%
1	117	27.7%
2	63	14.9%
3	13	3.1%
4	1	0.2%
	423	100.0%

The relationships among comorbidities were investigated using Spearman's correlation, along with other statistical groups. Statistically significant associations were found: A weak correlation was calculated between hypertensive disorders of pregnancy and mode of delivery (r = 0.295; n = 423; p < 0.005), indicating that mothers with hypertensive disorders of pregnancy more frequently had deliveries by cesarean section. A weak negative correlation was observed between hypertensive disorders of pregnancy and PROM (r = -0.220; n = 423; p = 0.00001), suggesting that mothers with hypertensive disorders of pregnancy were less likely to experience PROM. A weak negative correlation was observed between maternal obesity and PROM (r = -0.231; n = 423; p < 0.005), indicating that obese mothers were less likely to experience PROM. Combined associations of all comorbidities with other statistical groups were also investigated: A weak correlation was observed between the presence of comorbidities and mode of delivery (r = 0.148; n = 423; p = 0.002286), suggesting that mothers with comorbidities were more likely to have cesarean deliveries. A weak negative correlation was observed between the presence of comorbidities and PROM (r = -0.273; n = 423; p < 0.005), indicating that mothers with more comorbidities were less likely to experience PROM. These data are presented in Table 15.

**Table 15:** Spearman's correlation of comorbidities in relation to other statistical groups r: test statistic (correlation coefficient r); p: p-value; neonatal hospitalization: in days; mode of birth: natural (vaginal) birth or cesarean section; PROM: premature rupture of membranes; NICU: neonatal intensive care unit Statistical test used: Spearman's rank correlation. *Significant at p < 0.05. **Significant at p < 0.01

Comorbidity		Gestational group	Apgar one minute	Apgar five minutes	Duration of neonatal hospitalization	Mode of birth	PROM	NICU
Thyroid disorders in pregnancy	r	-0.04543	0.04833	0.01252	0.02313	-0.02811	-0.05287	-0.00710
	p	0.35132	0.32137	0.79732	0.63518	0.56424	0.27799	0.88425
Hypertensive disorders in pregnancy	r	-0.04020	-0.08415	-0.08641	0.08255	0.295**	-0.220**	-0.04110
	p	0.40951	0.08388	0.07586	0.08994	0.00000	0.00001	-0.04110
Diabetes mellitus	r	-0.05016	-0.05874	-0.09426	0.04667	0.04659	-0.07460	0.02137
	p	0.30341	0.22797	0.05272	0.33829	0.33915	0.12554	0.66114
Obesity	r	0.005128	-0.05906	-0.04748	-0.00908	0.011947	-0.231**	0.051006
	p	0.916247	0.225448	0.329944	0.852245	0.806464	0.00000	0.295278
All comorbidities	r	-0.04835	-0.05801	-0.08036	0.054124	0.148**	-0.273**	0.003706
	p	0.321157	0.233807	0.098835	0.266705	0.002286	0.00000	0.939418

Mothers with hypertensive disorders of pregnancy were more frequently delivered by cesarean section, accounting for 50 out of 68 (73.53%), compared to natural (vaginal) delivery at 18 out of 68 (26.47%), which was highly statistically significant (χ² = 36.8691; p < 0.005; df = 1; χ²sig = 3.841459). These data are presented in Table 16.

**Table 16:** Distribution of mothers with hypertensive disorders of pregnancy and mode of delivery ∑: sum of data from the specified rows Data are presented as numbers (percentages). Statistical test used: chi-square test. Significant at p < 0.05

Hypertensive disorders in pregnancy	Vaginal birth		Cesarean section		N		Test statistic	p-value
Yes	18	26.47%	50	73.53%	68	100.00%	36.8691	p<0.005*
No	234	65.92%	121	34.08%	355	100.00%		
∑	252	59.57%	171	40.43%	423	100.00%		

Mothers with hypertensive disorders of pregnancy presented with PROM less frequently, in 18 out of 68 (26.74%) of cases, compared to those without PROM, who accounted for 50 out of 68 (73.53%); this difference was highly statistically significant (χ² = 20.38277; p < 0.005; df = 1; χ²sig = 3.841459). These data are presented in Table 17.

**Table 17:** Prevalence of mothers with hypertensive disorders of pregnancy and premature rupture of membranes PROM: premature rupture of membranes Data are presented as numbers (percentages). Statistical test used: chi-square test. Significant at p < 0.05

Hypertensive disorders in pregnancy	PROM				N		Test statistic	p-value
	Yes		No					
Yes	18	26.47%	50	73.53%	68	100.00%	20.38277	p<0.005*
No	200	56.34%	155	43.66%	355	100.00%		
∑	218	51.54%	205	48.46%	423	100.00%		

Mothers with obesity presented with PROM less frequently, in 39 out of 117 (33.3%) of cases, compared to obese mothers without PROM, who accounted for 78 out of 117 (66.67%); this difference was highly statistically significant (χ² = 21.71124; p < 0.005; df = 1; χ²sig = 3.841459). These data are presented in Table 18.

**Table 18:** Prevalence of mothers with obesity and premature rupture of membranes PROM: premature rupture of membranes Data are presented as numbers (percentages). Statistical test used: chi-square test. Significant at p < 0.05

Obesity	PROM				N		Test statistic	p-value
	Yes		No					
Yes	39	33.33%	78	66.67%	117	100.00%	21.71124	p<0.005*
No	180	58.63%	127	41.37%	307	100.00%		
∑	219	51.65%	205	48.35%	424	100.00%		

**Figure 3:**
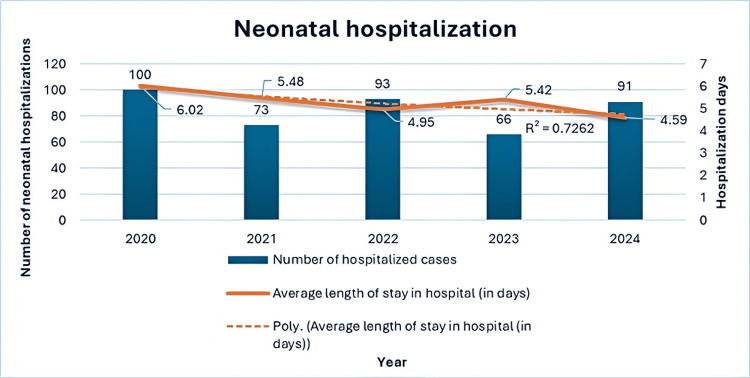
Neonatal hospitalization during the observed years

Neonates were, on average, hospitalized for 5.29 ± 3.61 days, with a minimum stay of one day and a maximum of 19 days. The median for the observed period was 5, and the mode was 3. When examining hospitalizations over the years, babies were, on average, hospitalized for the longest period in 2020 (6.02 ± 3.75 days) and for the shortest in 2024 (4.59 ± 3.04 days). These data are presented in Table 19 and Figure 3. The trend line for average neonatal hospitalizations in preterm births is decreasing, with a coefficient of determination of 0.7156.

**Table 19:** Duration of neonatal hospitalization in days by year

Year	N	Average	Standard deviation	Minimum	Maximum	Median	Mode
2020	100	6.02	3.75	1	17	5	3
2021	73	5.48	3.48	1	15	5	3
2022	93	4.95	3.79	1	19	5	1
2023	66	5.42	3.86	1	15	5	3
2024	91	4.59	3.04	1	14	4	1
∑	423	5.29	3.61	1	19	5	3

When observing the total and average length of hospitalization for women with and without comorbidities, it is noted that the average length of hospital stay for women with comorbidities, 5.57 ± 3.87 days, was higher than that for women without comorbidities, 5.06±3.37 days; however, this difference was not statistically significant (χ² = 0.81738; p = 0.3659; df = 1; χ²sig = 3.841459). These data are presented in Table 20.

**Table 20:** Duration of neonatal hospitalization in mothers with comorbidities Data are presented as mean (standard deviation). Statistical test used: chi-square test

Comorbidities	N	Total days	Average	Standard deviation	Test statistic	p-value
Yes	194	1080	5.57	5.06	0.81738	0.3659
No	229	1158	3.87	3.37		

Common associations between hospitalization duration and other variables were also investigated: a weak correlation was observed between hospitalization duration and mode of delivery (r = 0.117; n = 423; p = 0.017), suggesting that longer hospitalization duration was associated with a greater number of cesarean deliveries. A moderately strong negative correlation was observed between hospitalization length and NICU transfer (r = -0.535; n = 423; p < 0.005), with more extended hospitalization associated with a lower likelihood of NICU transfer. These data are presented in Table 21.

**Table 21:** Spearman's correlation of hospitalization days in relation to other statistical groups PROM: premature rupture of membranes; NICU: neonatal intensive care unit Statistical test used: Spearman's rank correlation. *Significant at p < 0.05. **Significant at p < 0.01

Hospitalization days	Gestational group	Apgar one minute	Apgar five minutes	Mode of birth	PROM	NICU	Mother age
Test statistic (correlation coefficient r)	-0.03827	0.01092	0.050038	0,117*	0.063791	-0,535**	0.069379
p-value	0.432	0.823	0.305	0.017	0.190	0.000	0.154

Common associations of NICU transfers with other statistical groups were also investigated: A moderately strong correlation was observed between NICU transfers and gestational age group (r = 0.483; n = 423; p < 0.005), indicating that NICU transfers were associated with earlier birth. A weak negative correlation was observed between NICU transfers and the one-minute Apgar score (r = -0.187; n = 423; p = 0.00011), suggesting that NICU transfers were associated with lower one-minute Apgar scores. A weak negative correlation was observed between NICU transfers and the five-minute Apgar score (r = -0.233; n = 423; p < 0.005), suggesting that NICU transfers were associated with lower five-minute Apgar scores. A weak negative correlation was observed between NICU transfers and PROM (r = -0.105; n = 423; p = 0.03), suggesting that NICU transfers were associated with a decrease in PROM. These data are presented in Table 22.

**Table 22:** Spearman's correlation of NICU transfer in relation to other statistical groups NICU: neonatal intensive care unit; PROM: premature rupture of membranes; mode of birth: natural (vaginal) birth or cesarean section Statistical test used: Spearman's rank correlation. *Significant at p < 0.05. **Significant at p < 0.01

NICU	Gestational group	Apgar one minute	Apgar five minute	Mode of birth	PROM	Mother age
Test statistic (correlation coefficient r)	.483**	-.187**	-.233**	0.015159	-.105*	-0.04474
p-value	0.00000	0.00011	0.00000	0.75589	0.03023	0.35870

## Discussion

This study analyzed the trends and determinants of preterm births at the Cantonal Hospital Zenica over a five-year period (2020-2024). The overall incidence of preterm birth in our study population was 3.80%, with no significant variations across the observed years. This rate is relatively low compared to global averages, which often range from 5% to 18% depending on the region [11]. Global epidemiological data suggest that preterm birth rates are heavily influenced by geographic, socioeconomic, and healthcare disparities, with significantly higher rates often reported in low-resource settings compared to European averages [12]. The consistency of the rate throughout the study period suggests a stable prevalence of risk factors in the Zenica-Doboj Canton, but it may also reflect the specific referral patterns of our institution as a secondary center, where high-risk pregnancies might be transferred antenatally, artificially lowering the local incidence [13].

Our analysis revealed that multiparous women accounted for a slightly higher proportion of the study population (53.19%) than primiparous women. Interestingly, while not statistically significant, there was a trend toward extremely preterm births (<33 weeks) being more common in multiparous women (75.0% vs. 25.0%). This aligns with established literature suggesting that a history of preterm birth is one of the most significant risk factors for recurrence in subsequent pregnancies [14]. Several studies indicate that the risk of recurrence is inversely related to the gestational age of the previous delivery; the earlier the previous birth, the higher the risk in the current pregnancy [14]. Conversely, we found a significant association between primiparity and PROM (52.75%). This is consistent with the hypothesis that nulliparity itself can be a risk factor for certain obstetric complications leading to preterm labor, possibly due to uterine distension anomalies or biological immaturity of the reproductive tract [15].

The most prevalent maternal comorbidity was obesity (27.4%), followed by hypertensive disorders of pregnancy (16.1%) and thyroid disorders (14.7%). Our study found significant correlations between these comorbidities and delivery outcomes. Increasing evidence points to maternal obesity as a state of chronic low-grade inflammation, which triggers a cascade of cytokines that can initiate preterm labor or lead to indicated deliveries due to metabolic complications [16]. Notably, women with hypertensive disorders were significantly more likely to undergo cesarean sections (73.53%) compared to vaginal deliveries. This high rate of surgical intervention is clinically expected, as severe preeclampsia or eclampsia often necessitates immediate delivery to safeguard maternal and fetal health, regardless of gestational age [17]. Current guidelines emphasize that the definitive treatment for severe hypertensive disorders is delivery, often precluding the option of expectant management to reach term [17]. Furthermore, thyroid dysfunction has been increasingly recognized as a determinant of adverse pregnancy outcomes, with thyroxine playing a critical role in placental development and maintenance of pregnancy [18].

An interesting finding was the negative correlation between maternal comorbidities (specifically hypertension and obesity) and PROM. This suggests that preterm births in these high-risk groups are more likely to be indicated (iatrogenic) due to maternal or fetal compromise rather than spontaneous (due to membrane rupture). This distinction between "spontaneous" and "provider-initiated" phenotypes is crucial for clinical management [19], highlighting the need for vigilant monitoring of hypertensive and obese pregnant women to time the delivery optimally.

Overall, vaginal deliveries were more frequent (59.6%) than cesarean sections (40.4%) among preterm births. However, the mode of delivery was heavily influenced by maternal pathology. As mentioned, hypertensive disorders strongly shifted the distribution toward cesarean sections [13,14]. Additionally, we observed that vaginal deliveries were significantly more common in cases complicated by PROM (64.68%). This likely reflects the clinical management of PROM, where induction of labor is often preferred over cesarean section if there are no other contraindications, to avoid surgical risks in the setting of potential infection (chorioamnionitis) [20]. While cesarean sections can be life-saving, they also carry higher risks of maternal morbidity compared to vaginal delivery, necessitating a balanced approach in preterm cases [21].

The majority of preterm neonates had favorable Apgar scores, though a slight, nonsignificant decline in one-minute scores was noted in specific years. A significant finding in our study was the correlation between NICU transfer and both gestational age and lower Apgar scores. This confirms that the most vulnerable neonates (those born earlier and with poorer immediate adaptation) are appropriately identified and transferred to the tertiary center in Sarajevo. This supports the effectiveness of regionalized perinatal care systems, where secondary centers stabilize and transfer high-risk neonates to specialized facilities to improve survival rates. The average length of neonatal hospitalization in our secondary center was relatively short (5.29 days), with a decreasing trend over the five years (R² = 0.7156). This decreasing trend, coupled with the finding that extended hospitalization correlates with fewer NICU transfers, suggests two potential patterns: either milder cases of preterm birth (late preterm infants) are being managed more efficiently at the secondary level, or the protocol for transferring neonates requiring prolonged care has become more streamlined. The fact that multiparous mothers had neonates with shorter hospital stays could imply better maternal experience and confidence in newborn care, facilitating earlier discharge readiness.

This study has several limitations. As a single-center retrospective study in a secondary referral center, the data may not fully capture cases of extremely high risk that might have been transferred antenatally to a tertiary center, potentially underestimating the true incidence of extreme preterm births in the region. Additionally, the lack of long-term neurodevelopmental follow-up data limits our ability to assess the full impact of preterm birth on these infants [22]. Despite these limitations, the study provides valuable epidemiological data for the region and highlights key targets for preventive interventions.

## Conclusions

This study at the Cantonal Hospital Zenica (2020-2024) revealed a stable, relatively low preterm birth rate of 3.80%. The research identified a dual etiology: spontaneous preterm labor, often associated with PROM in primiparous women; and medically indicated preterm births, strongly driven by maternal comorbidities like hypertensive disorders and obesity, leading to higher cesarean section rates. Neonatal outcomes featured favorable Apgar scores and a decreasing trend in hospitalization duration, with effective NICU transfer for the most vulnerable infants. The findings emphasize the critical need for targeted antenatal surveillance and management of modifiable maternal risk factors to improve perinatal outcomes.
